# How Does Mobile Workplace Stress Affect Employee Innovative Behavior? The Role of Work–Family Conflict and Employee Engagement

**DOI:** 10.3390/bs12010002

**Published:** 2021-12-22

**Authors:** Xinyuan Wang, Zhenyang Zhang, Dongphil Chun

**Affiliations:** 1 School of Economics and Management, Hulunbuir University, Hulunbuir 021000, China; wangxinyuan@pukyong.ac.kr; 2 Graduate School of Management of Technology, Pukyong National University, Busan 48547, Korea

**Keywords:** mobile workplace stress, work–family conflict, employee engagement, employee innovative behavior

## Abstract

The new wave of interest in mobile workplaces is profoundly changing the internal ecology of Chinese companies and creating new stress for employees. To investigate the mechanisms of mobile workplace stress on employee innovative behavior and the role of work–family conflict and employee engagement, we collected 426 valid samples from married male employees in the software and information service industries. The results show that mobile workplace stress has a significant negative effect on employee innovative behavior. In contrast, it has a significant positive effect on work–family conflict and employee engagement. In addition, work–family conflict partially mediates the relationship between mobile workplace stress and employee innovative behavior; employee engagement produces the suppressing effects. The chain intermediary effect of work–family conflict and employee engagement between the mobile workplace and employee innovative behavior is present. When we focus on the high performance of the mobile workplace, we should also pay attention to its impact on the company’s ability for innovation.

## 1. Introduction

Along with the rapid development of technology, the current mobile workplace is considerably different from the past. The all-in-one mobile workplace represented by DingTalk and WeCom profoundly changes how Chinese people work and live. As of January 2021, both apps have over 400 million registered users and over 100 million monthly active users. More and more companies require employees to install such apps on their smartphones to clock in and out, report to superiors, and contact customers. Some managers issue notices and assignments through the mobile workplace after work hours and require subordinates to check and respond within a short time. When the mobile internet was not widely used, although some companies would use email and SMS to deliver work messages to employees who were not at work [1], employees could choose to hold off on checking or responding to these notices or assignments. However, now, superiors can use these apps to see whether and when the messages that were sent are checked. When subordinates do not respond promptly, some apps will automatically convert text messages to voice messages and broadcast them to employees as a phone call.

Some studies show that using a mobile workplace has helped some companies to improve performance and reduce costs [2]. A mobile workplace can expand office space and time to suit new economic forms and lifestyles changed by COVID-19 [3], ensuring continuous and efficient business operations. While changing the internal ecology of companies, this trend is also shifts the stress of the traditional workplace to the mobile workplace [4], including increasing employee engagement to fulfill their job responsibilities, improving personal performance, and thus achieving organizational goals [5]. Although the mobile workplace can be stressful, as an office model innovation, it can promote employee engagement and thus promote innovative behaviors [6].

The introduction of a mobile workplace has also led to new problems for companies. The mandatory implementation of the mobile workplace in some companies has been resisted by employees, who have given these apps an abysmal rating in the app stores. Many dispute that mobile technology has blurred the boundaries between work and family, making it easier than ever for work to intrude into the family life of average employees [7], resulting in work–family conflict (WFC) [8]. Under the pressure of the ever-present mobile workplace, too much work–family conflict can make employees feel exhausted and lazy, reducing employee engagement [9]. We must realize that improving performance by increasing employee workload may increase more than employee stress. Some cases show that a failed management model for innovation could instead hurt a company’s innovation ability [10].

Employee innovation behavior is influenced by individual factors (e.g., employee engagement [11], work domain-related factors (e.g., organizational climate [12,13], leadership style [14]), and non-work domain-related factors (e.g., work–family conflict [15]). However, relatively few studies incorporate mobile workplace stress and consider multiple factors in an integrated manner, and this is a trend that cannot be ignored. Companies should focus on how to optimize organizational real and virtual environments to promote employee innovative behavior [16]. To provide theoretical support for company decisions, we try to explore the impact of mobile workplace stress on employee innovative behavior from the perspective of work–family conflict and employee engagement.

The remainder of this paper is structured as follows. Section 2 introduces the theoretical analyses and research hypotheses. Section 3 shows the research design, introducing the scale and items. Section 4 discusses the results that tested the measurement model and hypotheses. Section 5 concludes the paper and provides recommendations.

## 2. Literature Review and Hypotheses

### 2.1. Work–Family Conflict and Employee Engagement

Work–family conflict is a type of inter-role conflict [17]. Usually, employees are required to play different roles given by work and family at the same time. However, in reality, the stress from work and family are often incompatible, and they both compete for the employee’s limited time and energy [18]. The time and stress with the fulfillment of work requirements could interfere with fulfilling family-related responsibilities. This means that work–family conflict will arise when employee work and family demands are irreconcilably at odds [19]. It is generally accepted that work–family conflict could occur in two directions: work interferes with family (WIF), and family interferes with work (family–work conflict, FIW), which are interrelated but have distinct structures [20]. This study followed the research design and recommendations of Yun et al. (2012) [2] and Zhou et al. (2021) [21] by focusing only on how work affects the family (WIF) and not on how family affects work (FIW). This is because the hasty introduction of FIW in the absence of literature support is both inconsistent with the main idea of this study and may result in the findings becoming difficult to explain.

Employee engagement is considered as a positive attitude that employees hold toward the organization and its values [22]. Employee engagement reflects the physical, time, cognitive, and emotional investment in the performance of tasks [5], fostering work-related connections with others and thus contributing to the efficient operation of the business. Employee engagement is characterized by vigor, dedication, and devotion, which is persistent and pervasive [23]. Based on the social exchange theory, Saks (2006) [24] argued that employee engagement results from the reciprocal exchange between employees and companies.

Work–life balance is a must for all employees. Research has found that the dual demands of work and family lead to work stress and reduced engagement [25]. Karatepe et al. (2016) [26] used the work–family conflict as a mediator and examined the effect of person–job fit on employee engagement. From the perspective of emotional exhaustion, Liu et al. (2019) [27] studied the effect of work–family conflict on employee work engagement. Bian et al. (2019) [28] used the work–family conflict as a mediating variable and analyzed the effects of management support, time demands, and career concerns on employee work engagement. Accordingly, we predicted:

**Hypothesis** **1.** *Work–family conflict has a significant negative effect on employee engagement.*

### 2.2. Employee Engagement and Employee Innovative Behavior

A company’s innovation is an organic combination of all employee innovation. Long-term development cannot be separated from the leadership of the entrepreneur and management behavior, but it should also include the promotion of the employee innovative behavior [16]. Innovative behavior refers to the innovative ideas that employees generate at work to improve the organization’s current situation and implement them, usually with challenging, risky and complex characteristics [11]. The full expression of employee innovative behavior (EIB) comes from the study of Janssen (2005) [29], which focuses on the resistance and internal legitimacy issues that individual employees face when driving innovation. Following this, several scholars have interpreted it as job performance.

Some have argued that employee innovative behavior is an in-role performance and part of the employee job content [30]. In contrast, others [31] have argued that it is an extra-role performance because innovation is usually not a formal job requirement for employees. The former description is closer to the company’s R&D staff, while the latter could be extended to most employees. Kwon and Kim (2020) [32] argued that employee innovative behavior is a unique type of job performance. It is inaccurate to generalize employee innovative behavior by using the behavior of individual employees breaking explicit or implicit rules. Employees usually need some tacit understanding to establish an effective work communication mechanism to guarantee the sharing of knowledge and information, which also manifests employee innovative behavior. Once this set of rules is broken, employee innovative behavior will be affected.

In existing studies, employee innovative behavior is usually presented as a dependent variable. For example, Huang et al. (2016) [33] analyzed the positive impacts of employee perceived corporate employee responsibility on employee innovative behavior, using job satisfaction and job engagement as mediating variables. Companies with a high demand for innovation increasingly rely on highly dedicated employees who possess the ability to maintain an enthusiastic work attitude, consistent perseverance to work, a willingness to innovate, and a high sense of responsibility. With a high level of involvement, the employees are much more likely to think optimally about the solution to the problem and eventually show a high level of innovation [34]. This positive effect of employee engagement on employee innovation behavior is consistent with existing research [6]. Kim and Koo (2017) [35] analyzed the effect of leader–member exchange on innovative behavior using job engagement and organization engagement as mediating variables. They also concluded that job engagement significantly affects innovative behavior. Accordingly, we predicted:

**Hypothesis** **2.** *Employee engagement has a significant positive effect on employee innovative behavior.*

### 2.3. Work–Family Conflict and Employee Innovative Behavior

The relationship between role stress and other dependent variables has been the focus of academic research. Existing research [36] has shown that role stress is an important cause of work–family conflict. Stress at work can directly affect the work domain and cross-cuttingly affect the family domain [37]. Time and energy are necessary resources to secure innovation, and work–family conflict can take away such resources from individuals. According to the resource allocation theory [38], when an employee realizes that he does not have enough resources to play a role in one domain, he will tend to divert resources that belong to another domain, leading to work–family conflict [39]. According to Van and Jehn (2002) [40], stress reduces employee responsiveness to novelty. After being under more stress, employees are more inclined to follow the rules at work and neglect challenging tasks, especially creative work, due to a lack of resources such as time and energy. According to the study of [41], employee innovative behavior consists of three structured processes and procedures: innovation generation, promotion, and practice. If the stage is affected by adverse factors, it will affect the innovative behavior. Work–family conflict adversely affects employee work and life at uncertain times. Therefore, work–family conflict may have a certain degree of negative impact on employee innovative behavior.

There is little early literature on the relationship between work–family conflict and employee innovative behavior. In recent years, scholars have gradually focused on this topic. For example, Chen and Huang (2016) [42] used work–family conflict and employee innovative behavior as dependent variables for employee engagement. However, they did not further examine how work–family conflict would affect employee innovative behavior. Choi et al. (2018) [15] studied the impact of work–family conflict on employee innovative behavior, mediated by organizational commitment and job satisfaction. Unfortunately, this study did not produce a significant result. We believe the reason for this is that the sample they selected was not typical enough. Zhang et al. (2020) [43] verified the negative effect of work–family conflict on employee innovative behavior using emotional balance as a mediating variable as well as a sample of female knowledge workers. Song et al. (2020) [11] also conducted a study with a sample of knowledge workers and empirically illustrated that this group’s negative impact was stronger. Accordingly, we predicted:

**Hypothesis** **3.** *Work–family conflict has a significant negative effect on employee innovative behavior.*

### 2.4. Mobile Workplace and Mobile Workplace Stress

Yun et al. (2012) [2] described the changes brought about by smartphones as opening a new door. Previously, in the regular workplace, people could close the door to stay away from their colleagues. Even if there were no door or the door had to be opened, there was a geographical distance between home and company. However, in the era of the mobile workplace, smartphones are used for both family and work matters; thus, the door is completely gone.

In early literature, mobile work is similar to remote work [44]. Whether on a business trip using a laptop to reply to work e-mails or holding a remote meeting at home, as long as we leave a fixed workplace, it could be regarded as a kind of mobile work at that time [1]. While there is still much conceptual overlap between the two, and even a Wikipedia search for mobile work automatically redirects to remote work, people have realized their many differences, especially after the global experience of working from home. At present, the meaning of mobile in the mobile workplace has changed from geographic mobility relative to a fixed workplace to the use of mobile technology. As long as there is a mobile network, employees can perform mobile work in traditional workplaces. Unlike traditional office automation systems (OA) that run on PCs, mobile workplaces often include SNS, and some can communicate with people outside the company.

We use the concept of mobile workplace stress to describe the impact of mobile working. Although many scholars have studied the effects of mobile office systems on employee work, they mainly use various models similar to the TPB or TAM to analyze intention to use [45]. However, in general, ordinary employees do not have the power to choose whether or not to use it. They can only passively accept it. To sum up, stress may be a better choice when we want to study the employee perspective. Bang and Tak (2016) [46] developed the first scale to measure mobile workplace stress and supported that this stress exacerbates work–family conflict and work–leisure conflict. They believe that the sources of mobile workplace stress are multiple, such as private life intrusion, check compulsion, excess working hours, and quick-reply pressure.

As society changes, apps represented by DingTalk and WeCom have become the base form of an all-in-one mobile workplace in China. More and more companies are moving their approval processes to the mobile workplace. Even if employees are still in the company, they may need to complete various tasks via their smartphones. There are fewer opportunities for face-to-face communication between colleagues in some large companies. Even if all are on the same floor, people are more accustomed to communicating through an app than before. Therefore, it is not only after an employee returns home that the mobile workplace will impact stress.

Mobile workplace stress is a double-edged sword. While Yun et al. (2012) [2] only demonstrates that work overload from mobile working can directly exacerbate work–family conflict, they also demonstrate that increased productivity can mitigate work overload. The mobile workplace completely blurs the boundary between work and family [47]. The disruption of work to the family is exacerbated by the increased number of hours employees work [48]. Zhou et al. (2020) [21] confirmed that employee use of mobile technology during non-working hours impacts work–family conflict. Work tasks follow the smartphones into the home and form the mobile workplace stress. Employees are in a constant state of low work separation, and resource protection capabilities are in a lower state of protection. The stress consumes time and energy that would otherwise belong to the family, making it impossible for employees to perform their family duties effectively.

In addition, other studies [49] have shown that working with smartphones increases productivity and that employee engagement is closely related to job performance. At the same time, from the employee’s point of view, once they become accustomed to the mobile office, they always perceive mobile workplace stress, and it will be difficult for them to detach themselves from work psychologically [50,51]. Employees cannot help but work anywhere at any time. Therefore, mobile workplace stress is also likely to directly impact employee engagement.

There is little direct literature that we can refer to regarding the effect of mobile workplace stress on employee innovative behavior. The existing studies discussed the impact on employee innovation behavior more in terms of mobile work technology. Kim and Shin (2015) [52] examined the impact of a smartwork environment on organizational commitment and innovation behavior and found that a smartwork environment did not have a significant impact on organizational commitment, but the IT complexity and security risks of a smartwork environment had a positive impact on innovation behavior. We analyzed the main reasons for this: first, their study was conducted in 2015, when the mobile workplace was more of a novelty and challenge for many people rather than a stressor as it is now, and second, they chose a sample from the global financial service industry (GFSI), which is too industry specific. Our study focuses on mobile workplace stress, as we make bold assumptions. When the use of a mobile workplace changes from a novelty to programmed work, the “anytime and anywhere” structure will increase the burden of employees and turn into stress, which will eventually reduce the motivation of employees to innovate. Furthermore, the mobile workplace has disrupted employee home life and has changed the way they share knowledge and information, causing a direct impact on employee innovative behavior. When the effects of the factors are considered together, work–family conflict and employee engagement could mediate the process by which mobile workplace stress affects employee innovation. Accordingly, we predicted:

**Hypothesis** **4.** *Mobile workplace stress has a significant positive effect on employee engagement.*

**Hypothesis** **5.** *Mobile workplace stress has a significant positive effect on work–family conflict.*

**Hypothesis** **6.** *Mobile workplace stress has a significant negative effect on employee innovative behavior.*

## 3. Methods

### 3.1. Participants and Procedure

We collected the data by questionnaires. To better assess the impact of the current Chinese mobile workplace, the survey was targeted at married employees in the software and information service industries. This choice is mainly based on the following considerations. First, according to a previous study [36], compared to employees in other countries, Chinese employees tend to fulfill their family responsibilities by earning more money, and working harder is the primary way. They show more endurance in the face of stress from the company, which has a lower effect on work–family conflict. Given some examples of failure, we tend to choose an industry with a heavy workload. Second, workers in this industry often have received higher education, are engaged in knowledge-intensive labor, are more receptive to new products, and have a higher level of mobile working [11,43]. In addition, many people work in this industry, which facilitates the distribution and collection of questionnaires, and they are the ideal target group.

The questionnaires were first distributed to a software outsourcing company with which we have a partnership for pre-testing, mainly for grassroots employees, and a total of 46 samples were collected. Among these 46 employees, the oldest was a 32-year-old male, and the youngest was a 22-year-old female; 17 had a college diploma, 26 had a bachelor’s degree, and three had a master’s degree. During this period, we realized that the gender imbalance in this industry was more serious, with only seven females in the sample, two of them being accountants and one in HR. Although gender differences have been the focus of research on work–family conflict [53], it is not our concern at the moment. According to the study, employee perceptions of company requirements to use technology during off-hours may have different consequences for men and women [54]. To obtain better results, we further specified the respondents as male in the formal survey to exclude the effects caused by gender differences. The sample of females collected during the pre-test was excluded.

We commissioned a professional Chinese survey website called Credamo to collect data during the formal survey period, and they promised to guarantee the accuracy of the surveyed population and the authenticity of the data. We requested 400 valid samples. After further screening and the combining of the 39 male samples collected during the pre-test, the final valid sample size was 426. All participants were working in Jiangsu Province, which is one of the regions where China’s software and information services have been developing at a faster pace in recent years. As Table 1 shows, they were between 22 and 48 years, with 198 (46.48%) between 22 and 30 years, 141 (33.10%) between 31 and 40 years, and 87 (20.42%) at over 40 years; 66 (15.49%) had received an education at the master’s level, 238 (55.87%) had received an education at the bachelor’s level, and 122 (28.64%) had received an education at the college level.

### 3.2. Measures

To ensure the accuracy of the scale translation, we invited two teachers from the department of English to translate and back-translate the scales. All items were rated on a seven-point Likert scale ranging from 1 to 7. 1 means strongly disagree, and 7 means strongly agree.

#### 3.2.1. Mobile Workplace Stress

We adopted a thirteen-item scale developed by Bang and Tak (2016) [46] to measure mobile workplace stress. The items include many aspects, such as “I always have to pay attention to smartphone whether a new message comes” and “When I receive a message, I must reply immediately”. The source of this scale is a Korean language paper. Although the authors had given the scale in English, considering that the survey was performed in Korea, we asked a local Korean student to translate the scale into Korean by referring to the context of the paper. Then, we translated the Korean scale into Chinese. We compared it with the version translated directly from English and made some revisions.

#### 3.2.2. Work–Family Conflict

Work–family conflict was measured with a five-item scale developed by Netemeyer et al. (1996) [8]. An example of an item is “Things I want to do at home do not get done because of the demands my job puts on me”. Our study focused only on work interfering with family; thus, we did not use another scale on family–work conflict.

#### 3.2.3. Employee Engagement

We adopted a nine-item scale developed by Schaufeli et al. (2006) [23] to measure employee engagement. The items includes “When I get up in the morning, I feel like going to work” and “I get carried away when working”.

#### 3.2.4. Employee Innovative Behavior

We adopted a six-item scale developed by Scott and Bruce (1994) [41] to measure employee innovative behavior. An example item was “Promotes and champions ideas to others”.

## 4. Results

### 4.1. Measurement Model

We conducted a confirmatory factor analysis (CFA) of the model, and the results showed that the factor loading of each item ranged from 0.677 to 0.817, greater than 0.6 [34]. As Table 2 shows, in the various model fit indicators: chi-square/degrees of freedom (χ^2^/df) = 1.237, less than 3; comparative fit index (CFI) = 0.989, greater than 0.950; Tucker–Lewis index (TLI) = 0.988, greater than 0.950; root mean square error of approximation (RMSEA) = 0.023, less than 0.060; standardized root mean square residual (SRMR) = 0.041, less than 0.080; which indicated a good model fit [55].

The relevant data are shown in Table 3. The value of Cronbach’s alpha and composite reliability (CR) of each factor is greater than 0.7 [56,57], and the value of average variance extracted (AVE) is greater than 0.5. All correlation coefficients are smaller than the square root of AVE values to their right and above [58]. Thus, convergent validity and discriminant validity were supported. We also used Harman’s single factor test to avoid the common method bias (CMB). The results showed that the variance explained by the first factor is 19.238%, which is much lower than the recommended 40% [56], indicating that there is no need for concern regarding the CMB.

### 4.2. Hypotheses Test

**Figure 1:**
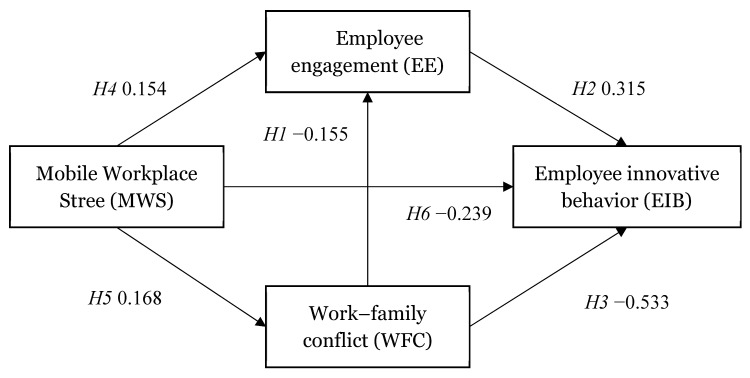
Results of structural equation model analysis.

We constructed a structural equation model to test the hypotheses, and the results are shown in Figure 1 and Table 4. Work–family conflict showed a significant negative effect on both employee engagement and employee innovative behavior. Hypotheses 1 and 3 were supported. Employee engagement significantly positively affects employee innovative behavior. Hypothesis 2 was supported. Mobile workplace stress showed a significant positive effect on both employee engagement and work–family conflict. Hypotheses 4 and 5 were supported. In addition, mobile workplace stress showed a significant negative effect on employee innovative behavior. Hypothesis 6 was supported.

Combined with the hypothesis test results, we further tested the mediating effect between the variables using Bootstrapping, with a sample size of 5000 and a confidence interval of 95%, although this might not have been necessary. The results showed that the total effect is −0.245 (CI: −0.336, −0.154); the direct effect of MWS on EIB is −0.212 (CI: −0.285, −0.139). Since the coefficient of the effect of MWS on WFC is a positive sign and the coefficient of the effect of WFC on EIB is a positive sign, the direct effect has the same sign as the indirect effect. The work–family conflict played a partial mediating role. Since the coefficient of the effect of MWS on EE has a positive sign, the coefficient of the effect of EE on EIB also has a positive sign, but the sign of the direct effect is opposite to that of the indirect effect. Thus, employee engagement played a suppressive role. Other data are shown in Table 5. Based on our search results, no one seems to have tested the mediating role of employee engagement in work–family conflict and employee innovative behavior. For this reason, we temporarily ignored mobile workplace stress and only tested the relationship between the other three variables. The results show that the total effect is −0.632 (CI: −0.720, −0.545), the direct effect is −0.599 (CI: −0.683, −0.515), and the indirect effect is −0.034 (CI: −0.056, −0.005). Employee engagement plays a mediating role between work–family conflict and employee innovative behavior.

The study then incorporated the effect of work–family conflict on employee engagement into the analysis in the form of a chain intermediary. The indirect effect of the path “MWS→WFC→EE→EIB” is −0.006 (CI: −0.009, −0.001), showing significance. WFC and EE act as a chain intermediary role between MWS and EIB.

## 5. Discussion and Conclusions

### 5.1. Discussion

Focusing on the stress from the new wave of the all-in-one mobile workplace, this study collected 426 valid samples from married male employees in the software and information service industries and constructed a structural equation model to analyze the impact of mobile workplace stress on employee innovative behavior in terms of work–family conflict and employee engagement.

The study found that mobile workplaces supplies convenience to companies and employees, but the mobile workplace also brings stress. This stress is ever present, blurring family boundaries and encroaching on employee energy and time, thus leading to work–family conflict (WFC).

Mobile workplace stress can increase employee engagement to some degree, which companies are hoping to achieve. As employee engagement increases, employees contribute more time and energy to work anywhere and anytime, and employee innovative behavior increases. However, this employee engagement is not unlimited, especially after work–family conflict arises, and employee engagement is naturally affected [2,21,27,28].

However, what may be unexpected to many entrepreneurs is that, at some point, the mobile workplace not only has a direct negative effect on employee innovative behavior, but it also further reduces them by exacerbating work–family conflict. While increased employee engagement with mobile workplaces can help to improve employee innovative behavior, it is still not enough to offset these harmful effects. Noteworthy among the results of our study is that between the mobile workplace stress and employee innovative behavior, work–family conflict and employee engagement play different roles individually. Work–family conflict acts as an intermediary, and employee engagement plays a suppressive role. A similar situation was found in some papers [15] closely related to this study, reflecting the phenomenon of “too much water drowned the miller”. Company decisions do not only produce a single effect. Considered as a whole, the chain intermediary effect of work–family conflict and employee engagement is present in the effect of mobile workplace stress on employee innovation behavior. This chain intermediary effect has been less used in studies of mobile workplace stress and is an innovative point in this study.

### 5.2. Practical Implications

There is no denying that the all-in-one mobile workplace has brought a whole new way of working. Compared to customizing their programs or systems, mobile workplace apps, with hundreds of millions of registered users, seem to provide a set of customizable digital control solutions for enterprises at a lower price. App developers tried to make entrepreneurs feel that overall efficiency improved and that everything seemed more organized. Companies and employees alike want to improve their innovation with the help of mobile workplaces. Nevertheless, what is criticized is that it also gives employees nowhere to hide in front of bosses, and the platform masters all employee actions at work. In the past, most company management software focused on controlling business by pursuing standardized processes and accurate data [2]. However, the all-in-one mobile workplace focuses on personnel, hoping to achieve business growth through accurate personnel control. Business owners need to think about how far they should apply such platforms and should carefully weigh the pros and cons.

There is nothing wrong with strengthening internal management, but it should never be at the expense of employee off-duty time. Long before the advent of all-in-one mobile workplaces, some European countries prohibited employers from using remote communication tools to contact employees after hours [46]. Today’s mobile workplaces are more severe in intensifying work–family conflicts than earlier traditional mobile communication methods such as SMS and email [1,4].

Companies could mitigate work–family conflict to avoid interrupting employees with unnecessary work after hours. It may require fostering a specific company culture and more restraint on management, especially to avoid using the mobile workplace as a tool to give orders anywhere, anytime. Specifically, companies need to establish more explicit rules to separate work from home activities and establish proper organizational communication protocols. The enterprise can formally code these rules as a technology use strategy. They can also be developed informally as part of the organizational culture.

Most employees are passionate about their companies and keep themselves together. In this case, companies should value their employees more and increase their work autonomy appropriately to maintain long-term stable employee engagement, such as reducing layers of approval in the approval system, giving employees a more relaxed workspace, more flexible working hours, and more accessible work boundaries.

We should see efficiency improvement and ignore those factors related to long-term development. The mobile workplace has changed the existing path of knowledge sharing and information transmission, and employee innovative behavior will be affected. To promote the mobile workplace, we need to unblock the internal communication pipeline in advance to avoid the failure to deliver ideas due to the reduced opportunities for employees to communicate face to face.

### 5.3. Limitations and Future Research Opportunities

Mobile office equipment tends to be convenient and fast, mobile office software tends to be fully functional and user-friendly, and the mobile workplace is the future of office trends. Mobile workplace stress is more complex and is worthy of research. Admittedly, this study was conducted in a single industry, a single gender, and a relatively close age of respondents, making it hard to apply the results to certain specific industries, especially those that have low technical requirements and only use such apps as a tool for clocking in and out of work. A previous study concluded that work–family conflict has a positive but small effect on employee innovative behavior [15]. We believe that this difference is related to our selected industry and the introduction of mobile workplace stress. It may instead illustrate the seriousness of the problems caused by the mobile workplace.

In recent years, some scholars have assessed the impact of the rapid development of mobile communication technologies on the intra-firm ecology from the perspective of technology overload [59], which provides ideas that can be used to continue to study this aspect in depth. As mentioned earlier, mobile workplace stress is a new research topic, and relatively few scales can be referred. In future research, we will continue to focus on developing mobile workplace stress in academic and managerial practice, conduct research in a more extensive population range and industry, and enrich relevant scales and findings.

Work–family conflict can be divided into work–family conflict and family–work conflict [8]. This study examines the only work–family conflict from an office workplace perspective. However, whether the family–work conflict impacts employee innovative behavior in the context of integrated mobile workplace applications is also worth studying.

## Figures and Tables

**Table 1:** The distribution characteristics of the sample.

Statistical Characteristics	Type	Frequency	Percentage (%)
Position	Product	83	19.48
	Operations	82	19.25
	Technology	108	25.35
	Marketing Others	92	21.6
	Administration	25	5.87
	Others	36	8.45
Education	The college level	122	28.64
	The bachelor level	238	55.87
	The master level	66	15.49
Age (year)	22–30	198	46.48
	31–35	96	22.54
	36–40	45	10.56
	>40	87	20.42

**Table 2:** Overall fit indices of the measurement model.

	χ^2^/df	CFI	TLI	RMSEA	SRMR
Scores	1.237	0.989	0.988	0.023	0.041
Criteria	<0.3	>0.9	>0.9	<0.05	<0.05

**Table 3:** Correlations, means, standard deviations, Cronbach’s alpha, composite reliability, and average variance extracted.

	1	2	3	4
1. Mobile workplace stress	***0.727***			
2. Work–family conflict	0.168	***0.716***		
3. Employee engagement	0.128	−0.129	***0.731***	
4. Employee innovative behavior	−0.288	−0.614	0.354	***0.838***
M	4.532	4.681	4.324	4.542
SD	1.057	1.016	1.048	1.147
α	0.849	0.879	0.889	0.942
CR	0.848	0.880	0.889	0.943
AVE	0.529	0.513	0.535	0.701

^1^ The bold italic diagonal elements are the square roots of each AVE, and the correlation coefficients are below the diagonal elements.

**Table 4:** Results of hypothesis testing through structural equation modeling.

Paths				Estimate	SE	Z	*p*	Std. Estimate	Results
H1	WFC	→	EE	−0.136	0.048	−20.810	0.005	−0.155	Supported
H2	EE	→	EIB	0.408	0.058	70.039	***	0.315	Supported
H3	WFC	→	EIB	−0.603	0.057	−100.524	***	−0.533	Supported
H4	MWS	→	EE	0.135	0.049	20.743	0.006	0.154	Supported
H5	MWS	→	WFC	0.169	0.056	30.024	0.002	0.168	Supported
H6	MWS	→	EIB	−0.272	0.050	−50.437	***	−0.239	Supported

^1^ *** *p* < 0.001.

**Table 5:** Test results of the two-factor analysis with causal mediation model.

Paths	Effect	Boot SE	BootLLCI	BootULCI	Z	*p*
MWS→WFC→EIB	−0.078	0.023	−0.120	−0.027	−3.321	0.001
MWS→EE→EIB	0.048	0.010	0.013	0.054	4.618	0.000
MWS→WFC→EE→EIB	−0.006	0.002	−0.009	−0.001	−3.005	0.003

## Data Availability

The data and models used during the study are available from the corresponding author by request.
